# Arterioureteral fistula after radical cystectomy and ureterocutaneostomy: two case reports and a systematic literature review

**DOI:** 10.1186/s12894-022-01071-y

**Published:** 2022-07-27

**Authors:** Zhiwen Jiang, Jian Wang, Jianfeng Cui, Shouzhen Chen, Sifeng Qu, Wenfu Wang, Hu Guo, Benkang Shi, Yaofeng Zhu

**Affiliations:** 1 Department of Urology, Qilu Hospital of Shandong University, Jinan, 250012 Shandong Province People’s Republic of China; 2 Department of Urology, People’s Hospital of Laoling, Laoling, Shandong Province People’s Republic of China

**Keywords:** Arterioureteral fistula, Literature review, Bladder cancer, Ureterocutaneostomy

## Abstract

**Background:**

Arterioureteral fistula (AUF) is a rare, life-threatening condition wherein communication occurs between a ureter and the common, internal, or external iliac artery. The sensitivity of common clinical imaging examination for AUF is low, which leads to a delayed diagnosis and increased mortality. In addition, the increased use of ureteral stents contributes to the growing frequency of AUF.

**Case presentation:**

Our two patients were 74 and 65 years old males respectively. They both had a medical history of bladder cancer and underwent radical cystectomy with ureterocutaneostomy. The patients underwent routine catheter exchange during over 1 year postradical cystectomy and subsequently experienced intermittent gross pulsatile haematuria. After a series of imaging examinations failed to identify the cause, the patients were ultimately diagnosed with AUF and treated with interventional radiotherapy, followed by broad-spectrum antibiotics. Positive effects were found.

**Conclusions:**

The incidence of AUF is increased with the prolongation of survival in patients with related risk factors. This case report aims to highlight early diagnosis and management of AUF to lower the mortality.

## Background

Arterioureteral fistula (AUF) is a rare but potentially life-threatening condition that was first reported in 1908 by Moschcowitz [1]. The pathophysiology of AUF involves the development of communication between a ureter and the common, internal, or external iliac artery. The causes of AUF can be divided into primary (15%) and secondary (85%) types [2]. Pelvic radiotherapy, genitourinary surgery, chronic ureteral stenting, and peripheral arterial disease are the most common secondary causes [3]. While haematuria is the most common symptom of AUF, flank pain, urinary retention, and infection have been described in the clinical as well [4]. Although AUF is uncommon, the mortality rate can reach 10% to 20% and increases in cases where the preoperative diagnosis is delayed [5]. We herein report two cases of AUF patients manifesting gross haematuria after radical cystectomy with ureterocutaneostomy.

## Case presentation

### Case 1

A 74-year-old male with persistent gross haematuria and flank pain was admitted to our department. He had a medical history of bladder cancer and asthma. In addition, he had severe obstructive pulmonary disease for many years. Bladder cancer was treated with radical cystectomy and ureterocutaneostomy in another hospital two years ago and the final pathology report revealed high-grade urothelial carcinoma of the bladder, stage unknown. A single-J polymeric stent was inserted and replaced every 3 months after surgery. However, a few days after the last replacement, the patient began to suffer from persistent gross haematuria and flank pain, without other significant symptoms. There was deep-red liquid and blood clots in the fistula bag. Contrast-enhanced computed tomography (CT) showed a low-density filling in the left renal pelvis. After admission, he developed haemodynamic instability and received 4 U red blood cell and 2 U haemocoagulase. After supportive treatment, the patient's symptoms were relieved significantly. The patient's urine became clear, and his vital signs gradually stabilized.

**Fig. 1:**
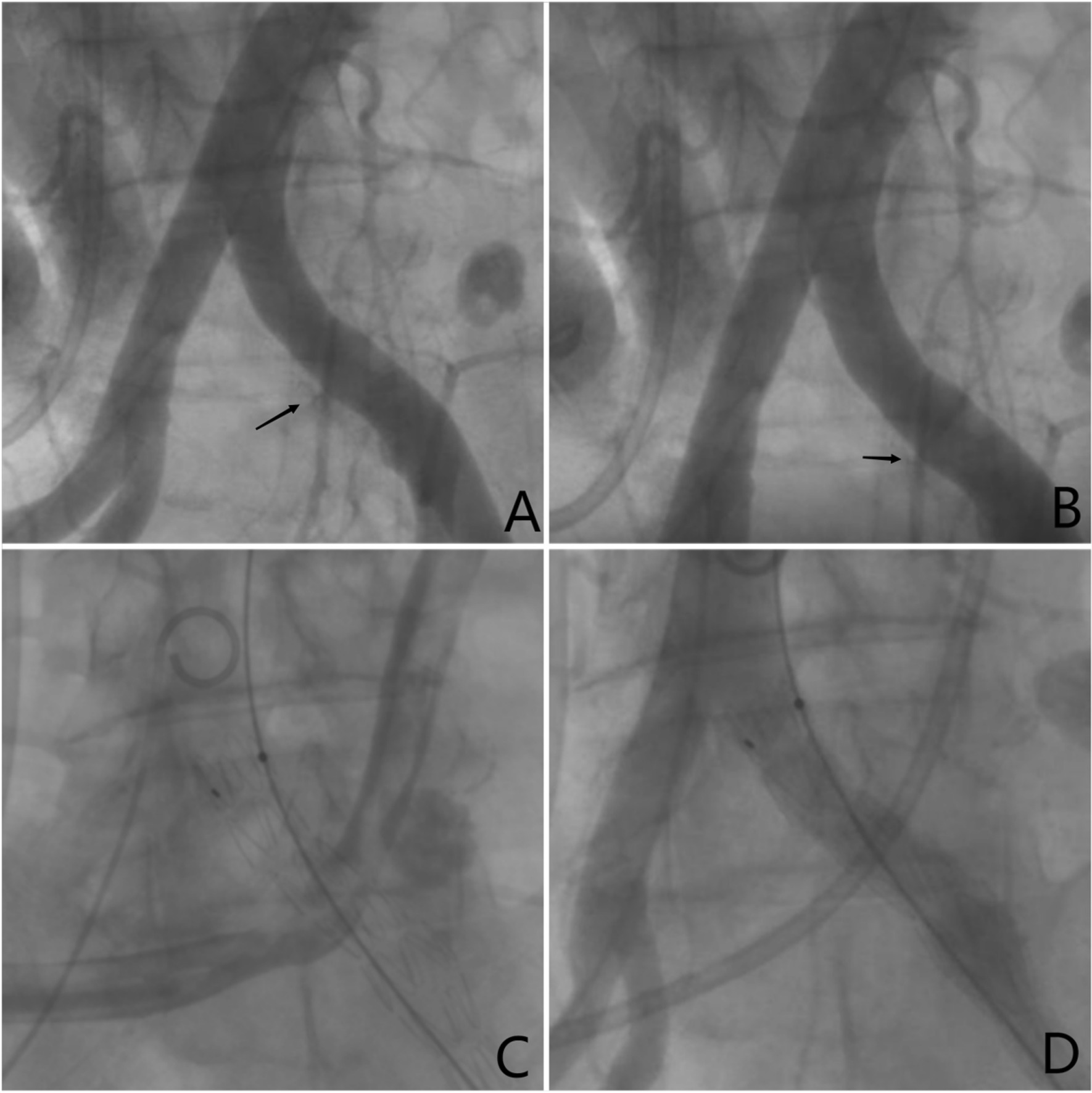
**A–B** Left arteriogram with contrast noted a exudation through the fistula tract from left iliac artery into the left ureter. **C–D** No contrast agent exudation was found in both arteriogram and nephrostogram after a heparinbonded stent-graft placed in left iliac artery

In the early morning of the third day of hospitalization, bright-red liquid and blood clots were noticed in the patient’s fistula bag. As the blood pressure (BP) is 90/60 mmHg and the heart rate is 90, his haemodynamic status was instable. Timely support treatment was given to maintain hemodynamic stability. Nonethless he continued to experience intermittent haematuria. The Interventional Radiology Department was urgently contacted, and emergent diagnostic catheter angiography was performed for suspected arterioureteral fistula. With the movement of contrast medium, a fistula was observed at the intersection of left ureter and common ipsilateral iliac artery. The patient was diagnosed with AUF (Fig. 1A–B); placement of a covered stent by endovascular treatment during interventional radiotherapy was immediately requested (Fig. 1C–D). The degree of haematuria gradually improved until disappearing and the patient was discharged 5 days after the operation with good diuresis and haemodynamic stability and remained free of gross haematuria during the 1-month follow-up. The patient was satisfied with the treatment results.

### Case 2

A 65-year-old male with intermittent haematuria was admitted to our department. He had a medical history of bladder cancer and accepted radical cystectomy with ureterocutaneostomy 15 months ago. Besides, He had a medical history of myocardial infarction and obstinate cardiac insufficiency. The patient’s final pathology report revealed high-grade urothelial carcinoma of the bladder, stage pT_2_N_0_M_0_. The patient exhibited no other discomfort except the haematuria in left single-J polymeric stent. His haemodynamic status was stable. Enhanced CT and magnetic resonance imaging (MRI) were performed, but there were no findings capable of explaining the patient's clinical symptoms. Combined with the patient's medical history of pelvic surgery and long-term ureteral stent implantation, AUF was suspected. Ureteroscopy and angiography were performed to assess fistula after the correction of anaemia. However, no bleeding point was identified clearly on angiography. Rough mucosa was found 15 cm from the ureteral orifice on ureteroscopy. Then, ureteral stent implantation was performed for urine drainage. No haematuria was detected during several days of hospitalization, and the urine drained by the ureteral stent was clear. The patient was discharged 5 days after the operation and followed up regularly.

**Fig. 2:**
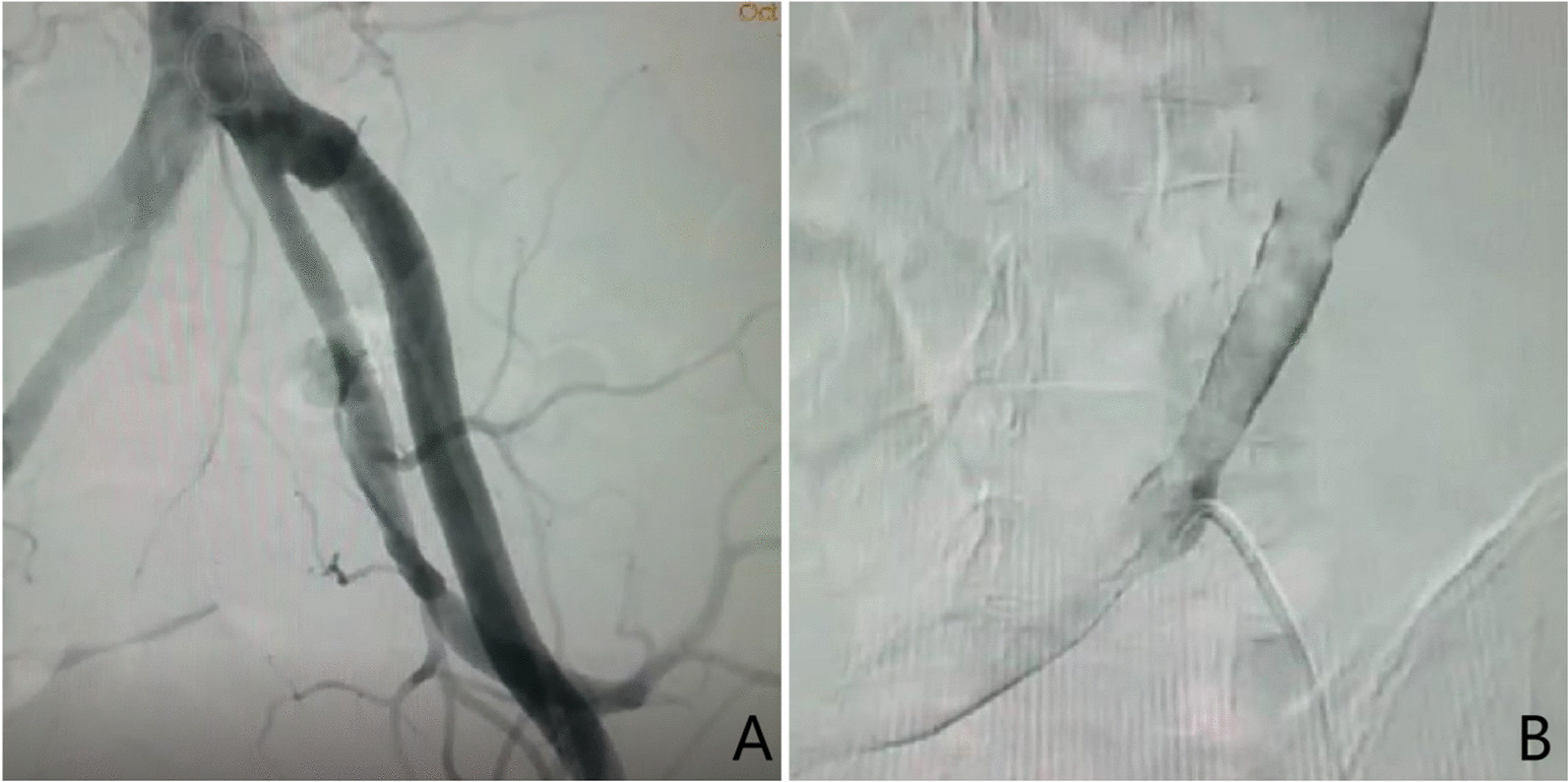
**A** Left arteriogram with contrast noted extravasating through the fistula tract. **B** The catheter entered the ureter through the fistula of the iliac artery

One month after returning home, the patient developed massive haematuria when the ureteral stent was replaced and accepted angiography in a local hospital. Fortunately, a fistula was found (Fig. 2A–B), and an iliac artery stent was placed. The patient was finally diagnosed with AUF. The patient's haematuria symptoms did not appear again during the 1 month of follow-up.

## Discussion and conclusion

The occurrence of AUF is not common, yet in the present era of increasing longevity and huge increases in accessibility to endoscopic interventions of the urinary tract, there is now a widespread recognition of AUF. In recent years, more than 150 cases of AUF with various causes have been reported [6]. Furthermore, AUF has become easier to diagnose due to the prolonged survival of patients with malignant tumours [7]. A review gave a summary of literature reports of 139 case reports of AUF from 1899 to 2008, in which gynaecologic cancer (28%), bladder cancer (13%), colorectal cancer (11%), other cancers (5%), untreated aneurysm (4%) and prior vascular surgery (18%) were mentioned [8]. Pregnancy-associated AUF was discussed in 3 reports [9]. Moreover, a study including 445 patients showed that 80% had chronic indwelling ureteral stents while 70% with a history of pelvic oncology, and most AUFs occurred at the common iliac artery ureteral crossing [10]. We reviewed 216 cases of 92 studies in English from the past 10 years (2011–2021) in PubMed and summarized them in Table 1. Risk factors included oncology (173 patients), ureteral stent placement (187 patients), radiotherapy (136 patients), aneurysm or pseudoaneurysm of the iliac artery (19 patients), vascular surgery (21 patients) and others (25 patients). The 213 patients in 92 studies consisted of 131 females and 82 males, with a mean age of 65.1 years (range 35–90 years). Details are provided in Table 2.

**Table 1:** Case reports review of past 10 years(2011–2021)

Publication date	Title	DOI	Cases	Age	Gender	Primary Disease (cases)	UAF Risk Factors (cases)	Symptoms	Diagnosis	Treatment
2021	Ureteroarterial fistula embolization by transradial approach: A case report	10.1016/j.radcr.2021.02.004	1	80	Female	Uterine cancer	U-stant, surgery, RT	Hematuria	Angiography	Embolism
2021	Ureteroarterial Fistula: A Diagnosis Which Is Not Always Black and White	10.1155/2021/8165991	2	55	Female	Cervical cancer	U-stant, surgery, RT	Hematuria	Retrograde Pyelography	Stent graft
				73	Female	Cervical cancer	U-stant, surgery, RT, Chemotherapy	Hematuria	No Clear Evidence	Open surgery
2021	Uretero-Arterial Fistula: A Case Report and Review of the Literature	10.1177/1538574420976731	1	56	Female	Ureteral stone	VS, laser lithotripsy	Hematuria	No Clear Evidence	Stent graft
2021	Oncology and complications	10.4081/aiua.2021.1.71	2	61	Male	Colon cancer	U-stant, surgery	Hematuria	Retrograde Pyelography	Stent graft
				63	Male	Retroperitoneal fibrosis	U-stant	Hematuria	No Clear Evidence	Stent graft
2021	A Bleeding Uretero-Arterial Fistula: Open Repair After Unsuccessful Endovascular Treatment	10.1177/1538574420953964	1	76	Female	Abdominal aneurysm	U-stant, VS, History of surgical injury to ureter	Hematuria	Angiography	Open surgery
2021	Management and endovascular therapy of ureteroarterial fistulas: experience from a single center and review of the literature	:10.1186/s42155-021–00,226-6	16	69.8	Male(12) Female(4)	Colorectal cancer (10) Uterine/cervical cancer (3) Bladder cancer (1) Testicular cancer (1) Prostate cancer (1)	U-stent (16) RT (14) Chemotherapy (14) Surgery (13)	Hematuria (16) Flank pain (5)	Enhanced CT (2) Angiography (3) No Clear Evidence(11)	Stent graft (10) Embolism (6)
2021	Endovascular therapy of arterioureteral fistulas	10.1024/0301–1526/a000922	5	64	Male(2) Female(3)	Pelvic malignancyt (4) Peripheral arterial disease (1)	U-stent (4) RT (4) Surgery (4)	Hematuria (5)	Enhanced CT (2) Angiography (5)	Embolism (5)
2021	Asynchronous Bilateral Ureteric-Arterial Fistula: Diagnosis and Treatment	10.1155/2021/5590432	1	48	Female	Cervical cancer	U-stant, surgery, RT, Chemotherapy	Hematuria	Enhanced CT Angiography	Stent graft
2021	Midterm Results after Open Surgical and Endovascular Management of Arterioureteral Fistula	10.1016/j.avsg.2020.11.014	9	69.1	Male(6) Female(3)	Pelvic malignancyt (6) Arterial disease (2) Aneurysm (1)	U-stent (7) RT (4) Surgery (9) VS (3)	Hematuria (9)	Ureteroscopy (3) Angiography (6)	Stent graft (4) Embolism (2) Open surgery (3)
2020	Ureteroiliac artery fistula caused by full-length metallic ureteral stenting in a malignant ureteral obstruction: a case report	10.1186/s13256-020–02,532-4	1	57	Female	Cervical cancer	U-stant, surgery, RT, Chemotherapy	Hematuria	No Clear Evidence	Stent graft
2020	Case Report of a Ureteroiliac Artery Fistula	10.1016/j.avsg.2020.09.026	1	63	Male	Rectal cancer	U-stant, surgery, RT, Chemotherapy	Hematuria	Angiography	Open surgery
2020	Ureteroarterial fistula treated by endovascular stent placement	10.1016/j.radcr.2020.05.044	1	69	Female	Cervical cancer	U-stant, surgery, RT	Hematuria	Angiography	Stent graft
2020	Ureteroarterial fistula: imaging diagnosis and endovascular management	10.1136/bcr-2020–236,011	1	65	Female	Cervical cancer	U-stant, surgery, RT, Chemotherapy, PA	Hematuria	Angiography	Stent graft
2020	Uretero-Iliac artery fistula: a rare cause of haematuria	10.1136/bcr-2019–232,189	1	45	Female	Cervical cancer	U-stant, surgery, RT, Chemotherapy	Hematuria	Retrograde Pyelography	Open surgery
2020	Arterioureteral Fistula in the Setting of an Indwelling Ureteral Stent, Ileal Conduit and History of Pelvic Radiation. Urology	10.1016/j.urology.2020.03.013	1	75	Female	Bladder cancer	U-stant, Cystectomy, RT, Chemotherapy	Hematuria	Retrograde Pyelography	Open surgery
2020	Uretero-arterial fistula treated with endovascular stent graft following multiple interventions	10.1002/iju5.12216	1	64	Male	Rectal cancer	U-stant, surgery, RT, Chemotherapy	Hematuria	Angiography	Embolism Stent graft
2020	Successful Endovascular Management of an Arterioureteral Fistula Presenting with Massive Hematuria in a Failed Renal Transplant	10.1089/cren.2019.0095	1	68	Female	Allograft renal Transplant secondary to chronic pyelonephritis	Allograft renal Transplant surgery	Hematuria	Angiography	Stent graft
2020	Endovascular treatment of arterio-ureteral fistula with new-generation balloon-expandable stent graft using a 7-French system	10.1177/2050313X20959219	1	82	Female	Cervical cancer	U-stant, surgery, RT, Chemotherapy	Hematuria	Enhanced CT	Embolism Stent graft
2020	Endoleak and Pseudoaneurysm Formation in the Setting of Stent Graft Infection Following Endovascular Uretero-Arterial Fistula Repair: The Dreaded Complication	10.7759/cureus.8830	1	71	Female	Cervical cancer Vaginal cancer	U-stant, surgery, RT, Chemotherapy	Hematuria	Angiography	Stent graft
2020	Endovascular and open surgical options in the treatment of uretero-arterial	10.1177/1708538120970823	25	61	Male(8) Female(17)	Endometrial adenocarcinoma (3) Cervical cancer (10) Prostate cancer (2) Bladder cancer (3) Aneurysm (2) Peripheral arterial disease (2) Colorectal cancer (2) Anal cncer (1)	U-stent (25) Surgery (21) RT (21) Aneurysm (2) Pelvic vascular bypass (2)	Hematuria (25) Flank pain (10)	Enhanced CT (7) Angiography (4) No Clear Evidence(14)	Stent graft (20) Open surgery (5)
2020	Ureteroiliac Fistula: Bleeding of Unknown Origin-Case Report and Review of the Literature	10.1089/cren.2020.0122	1	62	Male	Uncontrollable bladder bleeding	U-stant, surgery,RT	Hematuria	Ureteroscopy	Stent graft
2020	Clinics in diagnostic imaging (206)	10.11622/smedj.2020089	1	69	Female	Cervical cancer	U-stant, surgery, RT, Chemotherapy	Hematuria	Angiography	Stent graft
2020	Ureteral iliac artery fistula in idiopathic retroperitoneal fibrosis: A case report	10.4081/aiua.2020.2.107	1	73	Male	Retroperitoneal fibrosis	U-stant	Hematuria Flank pain	No Clear Evidence	Stent graft
2020	Arterioureteral fistula: overview of clinical characteristics, endovascular management, and outcomes	10.1080/13645706.2020.1782939	8	62.4	Male(2) Female(6)	Pelvic malignancyt (6) nephroure_x005fterectomy (1) Aneurysm (1)	U-stent (6) Surgery (7) RT (4) Aneurysm (1)	Hematuria	Enhanced CT (3) Angiography (8)	Stent graft (7) Embolism (1)
2020	Aorto-ureteric fistula post endovascular stent graft management of ruptured abdominal aortic aneurysm: a case report	10.1111/ans.15065	1	79	Male	Aneurysm	VS	Hematuria	Enhanced CT	Ureteric stent
2020	Life-threatening arterioureteral fistula treatment by endovascular complete anatomic iliac artery bifurcation reconstruction	10.1016/j.jvscit.2020.01.012	5	55	Male(1) Female(4)	Cervical cancer (4) Rectal cancer (1)	U-stent (5) RT (5) Chemotherapy (5) Surgery (5)	Hematuria	Angiography (5)	Stent graft (5)
2019	Endovascular treatment of ureteroarterial fistula using a covered stent, evaluated by intravascular ultrasound: a case report	10.1186/s42155-019–0060-6	1	84	Female	Retroperitoneal fibrosis	U-stant	Hematuria	Angiography	Embolism + Stent graft
2019	A Case of Ureteroarterial Fistula Successfully Treated with Endovascular Stent Graft	10.14989/ActaUrolJap-65–7-299	1	46	Female	Ovarian cancer	U-stant, surgery	Hematuria	Angiography	Stent graft
2019	Ureteroarterial Fistula in a Patient with an Ileal Conduit and Chronic Nephroureteral Catheter	10.1089/cren.2019.0004	1	64	Male	Bladder cancer	U-stant, Cystectomy	Hematuria	Angiography	Stent graft
2019	Endovascular management of arterio-ureteral fistula in a patient with a challenging hematuria. Minim Invasive Ther Allied Technol	10.1080/13645706.2018.1534742	1	43	Female	Cervical cancer	surgery, RT	Hematuria	Angiography	Embolism Stent graft
2019	Endovascular management and the risk of late failure in the treatment of ureteroarterial fistulas	10.1016/j.jvscit.2019.06.010	2	70	Female	Cervical cancer	U-stant, surgery, RT	Hematuria	Angiography	Stent graft
				77	Female	Cervical cancer	U-stant, surgery, RT	Hematuria	Angiography	Embolism Stent graft
2019	Sudden fatal bleeding from a uretero-arterial fistula combined with pre-existing uretero-colic and uretero-vaginal fistulas 7 years after a cervical cancer surgery: a case report	10.1186/s40792-019–0642-5	1	52	Female	Cervical cancer	U-stant, surgery, RT, Chemotherapy, Ureterocolic fistula and ureterovaginal fistula	Perineal hemorrhage	No Clear Evidence, Postmortem	Cannot accept intervention
2019	Ureteral-Arterial Fistula—A Role for Open Operation in the 21st Century	10.1016/j.jvs.2019.08.186	1	78	Male	Bladder cancer	U-stant, surgery	Hematuria	No Clear Evidence	Open surgery
2019	Endovascular management of arterio-ureteral fistula in a patient with a challenging hematuria	10.1080/13645706.2018.1534742	1	43	Female	Cervical carcinoma	U-stant, surgery, RT	Hematuria	Angiography	Embolism Stent graft
2019	The DACRON Ureter: A Case of Ureter to Aorto-Femoral Dacron Graft Fistulization	10.1016/j.urology.2018.11.005	1	65	Male	Peripheral artery disease	U-stent, VS	Hematuria	Angiography	Open surgery
2019	Ureteroarterial Fistula in a Patient with an Ileal Conduit and Chronic Nephroureteral Catheter	10.1089/cren.2019.0004	1	64	Male	Bladder cancer	U-stant, surgery	Hematuria	Angiography	Stent graft
2019	Uretero-iliac artery fistula: a challenge diagnosis for a life_x005fthreatening condition: monocentric experience and review of the literature	10.1007/s11255-019–02,097-2	3	66	Male(0) Female(3)	Cervical cancer	U-stant, surgery, RT	Hematuria	Angiography	Stent graft
2018	Iliac Artery-Uretero-Colonic Fistula Presenting as Gastrointestinal Hemorrhage and Hematuria: A Case Report	10.1089/cren.2017.0066	1	67	Female	Colon cancer	U-stant, surgery, RT, Chemotherapy	Hematuria	Enhanced CT	Embolism
2018	A rare complication of ureteral stenting: Case report of a uretero-arterial fistula and revision of the literature	10.4081/aiua.2018.3.215	1	79	Female	Endometrial carcinoma	U-stant, surgery	Hematuria	Angiography	Stent graft
2018	Uretero-arterial fistula due to a hypogastric aneurysm	10.1016/j.aju.2018.05.001	1	84	Female	Aneurysm	Aneurysm	Hematuria	Enhanced CT	Embolism Stent graft
2018	Two Cases of Arterioureteral Fistula in the Setting of Previous Radiation Therapy and Indwelling Ureteral Stents: Results of Endovascular Management	10.1016/j.clgc.2018.04.003	2	73	Female	Anal cancer	U-stant, PA, RT, Chemotherapy	Hematuria	Angiography	Embolism + Stent graft
				55	Female	Sigmoid adenocarcinoma	U-stant, surgery, RT, Chemotherapy	Hematuria	Angiography	Embolism Stent graft
2018	Diagnosis, Treatment, and Outcome of Arterioureteral Fistula: The Urologist’s Perspective	10.1089/end.2017.0819	26	67.9	Male(11) Female(13)	Endometrial adenocarcinoma (3) Cervical cancer (5) Vaginal cancer (2) Oophoroma (1) Peripheral arterial disease (2) Colorectal cancer (11) Metastatic carcinoma (2)	U-stent (26) Surgery (26) RT (21) Aneurysm (2) Pelvic vascular bypass (2)	Hematuria (24) Flank pain (11)	Enhanced CT (5) Angiography (9) No Clear Evidence(11)	Stent graft (23) Open surgery (3)
2018	Arterio-ureteric fistula: a rare but important cause of haematuria	10.1111/ans.14316	1	61	Female	Anal cancer	U-stant, surgery, RT, Chemotherapy	Hematuria	Angiography	Embolism
2018	Minimally invasive treatment of vascular complications after neoaortoiliac system reconstruction using autologous vein grafts	10.1016/j.jvscit.2018.08.013	1	54	Male	Aneurysm	U-stant, VS	Hematuria	No Clear Evidence	Stent graft
2018	Case—Uretero-internal iliac artery fistula presenting with multiple negative angiographic studies	10.5489/cuaj.4758	1	66	Female	Cervical cancer	U-stant, surgery, RT, Chemotherapy	Hematuria	No Clear Evidence	Embolism Stent graft
2018	Successful Endovascular Management of a Transplant Renal Artery Pseudoaneurysm Complicated With Arterioureteral Fistula	10.1177/1526924817746913	1	57	—	Post kidney transplantation	U-stant, surgery	Hematuria	Angiography	Stent graft
2017	Arterioureteral Fistula: Treatment of a Hemorrhagic Shock with Massive Hematuria by Placing a Balloon Catheter	10.1155/2017/9453618	1	52	Female	Colon cancer	U-stant, surgery, Chemotherapy	Hematuria Flank Pain	Enhanced CT	Stent graft
2017	Uretero-Arterio-Enteric Fistula Formation and Stent Thrombosis After Endovascular Treatment of Ureteroarterial Fistula: A Case Report and Review of Literature	10.1089/cren.2017.0108	1	51	Female	Cervical cancer	U-stant, surgery, RT, Chemotherapy	Hematuria	No Clear Evidence	Stent graft Open surgery
2017	Uretero-iliac artery fistula eight years after open abdominal aneurysm repair: A diagnostic and therapeutic challenge	10.1177/2051415816677502	1	79	Male	Aneurysm	U-stant, surgery, PA	Hematuria Flank pain	Enhanced CT	Stent graft
2017	Endoureteral coil embolization of an ureteral arterial fistula	10.1177/1708538117704522	1	38	Female	Postnephrectomy	U-stant, surgery	Hematuria Abdominal pain	No Clear Evidence	Embolism of ureter
2017	Iliac Artery-Uretero-Colonic Fistula Presenting as Gastrointestinal Hemorrhage and Hematuria: A Case Report	10.1089/cren.2017.0066	1	67	Female	Colon cancer	U-stant, surgery, Chemotherapy	Hematuria	Angiography	Embolism
2017	Ureteroarterial Fistulas: Diagnosis, Management, and Clinical Evolution	10.1016/j.avsg.2017.05.001	5	68	Male(3) Female(2)	Aneurysm (1) Aorto-iliac bypass (1) Bladder cancer (1) Rectal cancer (1) Ovarian cancer (1)	U-stent (4) Surgery (3) VS (2) Chemotherapy (2)	Hematuria (5)	Enhanced CT (3) Angiography (1) No Clear Evidence(1)	Stent graft (1) Embolism (2) Open surgery (2)
2017	Balloon-Expandable Stent Graft for Treating Uretero-Iliac Artery Fistula	10.1007/s00270-017–1586-4	8	64.5	Male(3) Female(5)	Pelvic malignancyt (8)	U-stent (7) Surgery (6) RT (5)	Hematuria (8)	Enhanced CT (2) Angiography (8)	Stent graft (8)
2016	Ureteroiliac Artery Fistula Caused by a Metallic Memokath Ureteral Stent in a Radiation-Induced Ureteral Stricture	10.1089/cren.2016.0097	1	71	Male	Colon cancer	U-stant, surgery, RT, Chemotherapy	Hematuria Abdominal Pain	Angiography	Stent graft
2016	Bilateral ureteroarterial fistula: a case report and review of literature. Urologia	10.5301/uro.5000164	1	50	Female	Endometrial carcinoma	U-stant, surgery, RT, Chemotherapy	Hematuria	Angiography	Embolism
2016	Unique Presentation of Hematuria in a Patient with Arterioureteral Fistula	10.1155/2016/8682040	1	54	Female	Cervical cancer	surgery, RT	Hematuria	Angiography	Embolism
2016	Arterioureteral fistula: an unusual clinical case	10.1136/bcr-2016–214,400	1	66	Male	Infection of the vascular graft	VS, Graft infection	Hematuria	Angiography	Open surgery
2016	Diagnosis and Management of a Challenging Patient: Ureteroarterial Fistula	10.1016/j.urology.2016.07.017	1	62	Female	Cervical cancer	U-stant, surgery, RT, VS	Hematuria	Retrograde Pyelography	Stent graft
2016	Ureteroarterial Fistulas After Robotic and Open Radical Cystectomy	10.1089/cren.2015.0034	2	82	Male	Bladder cancer	U-stant, Cystectomy	Hematuria	Angiography	Stent graft
				88	Male	Bladder cancer	U-stant, Cystectomy	Hematuria Abdominal pain	Angiography	Stent graft
2016	Management Strategy for Ureteral-Iliac Artery Fistula	10.1016/j.avsg.2016.02.033	6	61.7	Male(2) Female(4)	Rectal cancer (2) Cervical cancer (2) Bladder cancer (1) Ureteral stricture (1)	U-stent (6) Surgery (5) RT (3)	Hematuria	Enhanced CT (4) Angiography (2) No Clear Evidence(2)	Stent graft (2) Open surgery (4)
2016	Endovascular Repair of an Iliac Ureteroarterial Fistula with Late Stent Thrombosis and Migration into the Bladder	10.1016/j.avsg.2016.01.026	1	37	Female	Cervical cancer	U-stant, RT, Chemotherapy	Hematuria	Angiography	Stent graft
2016	Ilio-ureteric Fistula: A Rare Cause of Haematuria	10.1016/j.ejvs.2016.05.021	1	76	Female	Aneurysm	VS, PA	Hematuria	Enhanced CT	Embolism Stent graft
2016	Uretero-arterial fistula due to a hypogastric aneurysm	10.1016/j.aju.2018.05.001	1	84	Female	—	Aneurysm	Hematuria	Enhanced CT	Embolism Stent graft
2015	Ureteroarterial fistula following retrograde ureteral stenting in a patient with a double-barreled wet colostomy for cervical cancer	10.1016/j.gore.2015.06.007	1	64	Female	Cervical cancer	U-stant, surgery, RT, Chemotherapy	Hematuria	No Clear Evidence	Embolism Stent graft
2015	Successful endovascular treatment using a covered stent for artery_x005fureteral fistula after surgery for abdominal aortic aneurysm	10.4103/0970–1591.159668	1	63	Male	Aneurysm	U-stant, VS	Hematuria	No Clear Evidence	Embolism Stent graft
2015	Bilateral Ureteral-Iliac Artery Fistula in a Patient with Chronic Indwelling Ureteral Stents: A Case Report and Review	10.1155/2015/826760	1	58	Female	Cervical cancer	U-stant, surgery, RT	Hematuria	Angiography	Stent graft
2015	A rare cause of massive haematuria: Internal iliac artery-ureteric fistula	10.1177/1708538114538623	1	82	Male	Aneurysm	Aneurysm	Hematuria	Enhanced CT	Embolism Stent graft
2015	Complications after polymeric and metallic ureteral stent placements including three types of fistula	10.1089/end.2014.0394	3	64.7	Male(0) Female(3)	Cervical cancer (2) Rectal cancer (1)	U-stant, surgery, RT	Hematuria	Enhanced CT (1) Angiography (2)	Open surgery (3)
2015	Iliac Artery-Uretero-Colonic Fistula Presenting as Severe Gastrointestinal Hemorrhage and Hematuria: A Case Report and Review of the Literature	10.1016/j.avsg.2015.07.006	1	35	Male	Aneurysm	VS	Hematuria Hematochezia	No Clear Evidence	Stent graft Open surgery
2014	Ureteroarterial fistula from ureteral stump: a challenging case	10.1155/2014/514625	1	43	Female	Cervical cancer	U-stant, surgery, RT, Chemotherapy	Hematuria	Angiography	Embolism Stent graft
2014	Endovascular management of ureteroarterial fistula: a rare potentially life threatening cause of hematuria	10.3941/jrcr.v8i7.1879	1	70	Female	Uterine cancer	U-stant, surgery, RT, Chemotherapy	Hematuria	Angiography	Stent graft
2014	Lessons learned from endovascular management of ureteroarterial fistula. Vasc Endovascular Surg	10.1177/1538574413510620	1	76	Female	Uterine cancer	U-stant, surgery, RT	Hematuria	Angiography	Embolism Stent graft
2014	Endovascular treatment of arterio-ureteral fistulae with covered stents: Case series and review of the literature	10.1177/2050313X14548094	2	44	Female	Cervical cancer	Surgery, RT, Chemotherapy	Hematuria	Angiography	Stent graft
				71	Female	Cervical cancer	U-stant, surgery, RT, Chemotherapy	Hematuria	Angiography	Stent graft
2014	Ureteroarterial fistula from ureteral stump: a challenging case	10.1155/2014/514625	1	43	Female	Cervical cancer	U-stant, surgery, RT, Chemotherapy	Hematuria	Angiography	Stent graft
2014	Diagnosis and treatment of arterial-ureteric fistula	10.1016/j.jvs.2013.06.015	1	45	Female	aorto-bi-iliac bypass	U-stant, VS, PA	Hematuria Flank pain	Angiography	Stent graft
2014	Lessons learned from endovascular management of ureteroarterial fistula	10.1583/04–1496.1	1	76	Female	Cervical cancer	U-stant, surgery, RT	Hematuria	Angiography	Stent graft
2013	Ureteroarterial fistula	10.1016/j.jvs.2011.12.050	1	54	Male	Infection of the vascular graft	U-stant,VS	Hematuria	Angiography	Stent graft
2013	Delayed massive hemorrhage due to external iliac artery pseudo-aneurysm and uretero-iliac artery fistula following robotic radical cystectomy and intracorporeal Studer pouch reconstruction: Endovascular management of an unusual complication	10.5489/cuaj.170	1	54	Male	Bladder cancer	U-stant, PA, Cystectomy	Hematuria	Angiography	Stent graft
2013	Uretero-iliac fistula: modern treatment via the endovascular route	10.1016/j.diii.2012.10.005	2	56	Female	Cervical cancer	Surgery, RT, Chemotherapy	Hematuria	Angiography	Stent graft
				59	Male	Colon cancer	U-stant, surgery, RT, Chemotherapy	Hematuria	Enhanced CT	Embolism Stent graft
2013	Successful endovascular treatment of iliac arteriovesical fistula with secondary stent-graft infection	10.1016/j.jvir.2013.05.047	1	58	Female	Uterine cancer	U-stant, surgery, RT, PA	Hematuria	Enhanced CT Angiography	Stent graft
2013	Long-term results of endovascular stent graft placement of ureteroarterial fistula. Cardiovasc Intervent Radiol	10.1007/s00270-012–0534-6	11	72.8	Male(4) Female(7)	Pelvic malignancyt (9) Retroperitoneal fibrosis (1) Aneurysm (1)	U-stent (10) Surgery (8) RT (5) Aneurysm (1)	Hematuria	Enhanced CT (3) Angiography (5) No Clear Evidence(5)	Stent graft
2013	Massive hematuria and shock caused by ilio-ureteral fistula in a patient with an isolated internal iliac artery aneurysm	10.3400/avd.cr.12.00066	1	73	Female	Aneurysm	Aneurysm	Hematuria	No Clear Evidence	Open surgery
2013	A primary arterial-ureteral fistula after an aortic-bifemoral bypass	10.1016/j.ijscr.2012.09.010	1	74	Male	Aortic-bifemoral bypass	VS	Hematuria	Angiography	Open surgery
2013	Ureteroiliac fistula secondary to radiotherapy in a patient with single renal metastasis of colon adenocarcinoma	10.5489/cuaj.259	1	61	Male	Rectal cancer	Surgery, RT, Chemotherapy	Abdominal pain	Enhanced CT	Open surgery
2013	Endovascular approach in a secondary arterio_x005fureteral fistula	10.1024/0301–1526/a000237	1	67	Male	Peripheral arterial disease	U-stent, VS, PA	Hematuria Flank pain	Enhanced CT	Stent graft
2013	A case of gross haematuria due to an ureteric-iliac artery fistula	—	1	75	Male	Aneurysm	U-stent, VS	Hematuria	Enhanced CT	Stent graft
2012	Ureteroarterial fistula	10.3109/01443615.2012.690788	1	42	Female	Cervical cancer	U-stant, surgery, RT, Chemotherapy	Hematuria	Angiography	Embolism Stent graft
2012	An unusual cause of aortofemoral bypass infection	10.1080/00015458.2012.11680799	1	70	Female	PA	U-stant, VS	Hematuria	Introoperative finding	Open surgery
2012	Uretero-iliac artery aneurysm fistula: A rare but fatal cause of haematuria	10.1093/jscr/2012.8.16	1	90	Male	Colon cancer	Bilateral iliac aneurysms	Hematuria pain	Postmortem	Cannot accept surgery
2012	Treatment of ureteroarterial fistula with an endoureteral stent graft	10.1016/j.jvir.2012.06.020	1	76	Female	Bladder cancer	U-stant, RT, Cystectomy	Hematuria	Angiography	Stent graft
2012	Uretero-internal pudendal artery fistula with longterm indwelling of ureteral stent: a case report	10.1155/2012/817942	1	74	Female	Cervical cancer	Surgery, RT, U-stent	Hematuria	Angiography	Embolism
2011	Endovascular treatment of a right-sided ureteroiliac fistula in a patient with a simultaneous left-sided ureteroileal fistula	10.1155/2011/284505	1	80	Female	Liver metastasis	U-stant, surgery, RT, Chemotherapy	Hematuria	No Clear Evidence	Stent graft

*CT*, computed tomography; *U-stent*, ureteral stent; *RT*, radiotherapy; *PA*, pseudoaneurysm; *VS*, vascular surgery

**Table 2:** Study group characteristics and risk factors for AUF

Characteristics	Value
Patients (n)	213
Publication date	2011–2021
Male sex (%)	38.5
*Age*	
Mean	65.1
Range	35–90
*Medical history*	
Oncology (radiotherapy/surgery/both)	173
Ureteral stent	187
Radiotherapy	136
Aneurysm or pseudo-aneurysm of iliac artery	19
Vascular surgery	21
Others	25

The pathophysiology of the formation of arterioureteral fistula is still unclear. However, it can be divided into primary iliac AUF and secondary iliac AUF according to medical history and the disease process. Primary AUF is mainly caused by aneurysm or pseudoaneurysm rupture and is associated with atherosclerosis or vascular surgery history [11]. In secondary AUF, radiotherapy and chronic ureteral stents might be risk Factors [6]. Changes in the media and adventitial layers of the large vessels are caused by prior radiation, rendering the tissues more prone to rupture, erosion and necrosis [12]. Besides long-term compression of the ureteral stent leads to tissue necrosis and fistula between the ureter and iliac vessels. Therefore, a ureteral stent replaced after the operation should be as soft and thin as possible. To prevent strong compression and abrasion of the ureteral tube wall, it is recommended that the stent be less than Fr8 [12]. If the stent tube is too hard, the ureter wall and the iliac artery, particularly the turning point of the ureter, will be under excessive pressure and forced close to the artery. In this way, a fistula can easily form under the erosion of the pulsating artery against the baseline mechanical friction caused by the pulsatile arterial flow [6]. Due to the limited fat support between the ureter and iliac vessels for patients with low BMI, this situation may also result in AUF. In patients undergoing lymph node dissection, the iliac vascular sheath is opened, exposing the vascular wall and further leading a lack of tough connective tissue protection between the iliac vascular sheath and the left ureter. The combined action might lead to fistula formation.

The most common symptom is haematuria, occasionally with flank pain. The degree of haematuria can range from intermittent bleeding to life-threatening haemorrhagic shock. In some cases obstructed clot formation in the ureter causes flank pain [2].

Although the danger of AUF has been mentioned in many studies, some patients cannot receive prompt treatment because of the difficulty of diagnosis. The most effective diagnostic method is digital subtraction angiography, yielding a diagnosis of 69% of 139 cases [8]. Angiography with concurrent manipulation of a neophroureteral stent has been shown to improve the sensitivity to 100%, which can achieve the same effect as balloon stimulation. The sensitivity of provocative retrograde pyelogram may be as low as 63% [13, 14]. Enhanced CT has less sensitivity in identifying bleeding but can be highly beneficial if active haemorrhage or a pseudoaneurysm is exist. It can also be used to rule out renal haemorrhages and plan endovascular treatment [15]. Cystoscopy may contribute to localized bleeding of the ureter.

There are two main therapeutic approaches to AUF: open surgery and endovascular treatment. Since 1996, a stent graft has been used as treatment for AUF in most patients [16]. Because of its minimally invasion and outcomes similar to those of open surgical reconstruction, endovascular treatment has become an appealing alternative to open surgical reconstruction [17]. It is important to note that intravascular stents increase the possibility of infection, which is an main factor leading to the shortening of postoperative survival [18]. Accordingly, empiric broad-spectrum antibiotics with the ability to penetrate bacterial biofilms should be used [18]. When replacing the ureteral stent regularly, use of a guide wire and a balloon with an appropriate amount of water in replacement of the ureteral stent can reduce the occurrence of AUF.

Regarding to our two cases with history of chronic ureteral stenting after radical cystectomy and ureterocutaneostomy, the priority of diagnosing AUF needs to be emphasized for patients with a history of pelvic malignancy, chronic ureteral stenting, pelvic irradiation or symptoms that include haematuria, flank pain, or both. Thus, the interval between the onset of symptoms and rapid progression of AUF may be minimized, which makes it reasonable to carry out emergency intervention without definitive imaging evidence in patients with related risk factors. The delay in clinical diagnosis may lead to the deterioration of the condition, which cannot be treated [12]. Overall, treatment results may be improved by timely angiography.

AUF is a life-threatening condition that can occur in patients with long-term ureteral stents. Although rare, AUF should be highly suspected if a patient has a medical history of pelvic surgery or pelvic irradiation in the setting of ureteral stents and haematuria. Timely interventional radiotherapy can help lower mortality.

## Data Availability

Not applicable.

## Abbreviations

AUF: Arterioureteral fistula
CT: Contrast enhanced computed tomography
